# Ethanol Extract of *Perilla frutescens* Suppresses Allergen-Specific Th2 Responses and Alleviates Airway Inflammation and Hyperreactivity in Ovalbumin-Sensitized Murine Model of Asthma

**DOI:** 10.1155/2015/324265

**Published:** 2015-04-20

**Authors:** Miaw-Ling Chen, Chi-Heng Wu, Li-Shiuan Hung, Bi-Fong Lin

**Affiliations:** ^1^Department of Nutrition and Health Sciences, College of Health Sciences, Chang Jung Christian University, No. 1, Changda Road, Guiren District, Tainan City 71101, Taiwan; ^2^Department of Biochemical Science and Technology, College of Life Science, National Taiwan University, Sec. 4, No. 1 Roosevelt Road, Taipei 10617, Taiwan

## Abstract

This study was to investigate the effects of different fractions of *Perilla frutescens* (Pf) leaves extracted by water or ethanol on asthma. BALB/c mice sensitized intraperitoneally and challenged with ovalbumin (OVA) were divided into six groups. Each group of mice was tube-feeding with 0 (control), 80 *μ*g (PfWL), or 320 *μ*g (PfWH) water extracts or 80 *μ*g (PfEL) or 320 *μ*g (PfEH) ethanol extracts of perilla leaves daily for 3 weeks. A negative control group (PBS) was neither sensitized nor treated with Pf. The effects of perilla leave extracts on allergic immune response were evaluated. The results showed that OVA-specific IL-5 and IL-13 secretions from OVA-stimulated splenocytes were significantly suppressed in the ethanol extract groups PfEL and PfEH. Serum level of anti-OVA IgE tended to be lower in the PfEH group. The inflammatory mediators, such as eotaxin and histamine, and total cells, particularly eosinophils in bronchoalveolar lavage fluid (BALF), were also decreased in the PfEL and the PfEH groups. Therefore, the PfEL and the PfEH groups had significantly lower methacholine-induced hyperresponsiveness (AHR). In conclusion, ethanol extracts, rather than water extract, of perilla leaves could significantly suppress Th2 responses and airway inflammation in allergic murine model of asthma.

## 1. Introduction

Allergic asthma is a chronic disease that clinically augments bronchial hyperresponsiveness and inflammation. The asthmatic inflammation is clearly associated with the high level of type 2 T cell (Th2) cytokines that induced immunoglobulin (Ig) E production and eosinophilic infiltration [1]. The Th2 cytokines IL-4, IL-5, and IL-13 are the major cytokines for development of atopic diseases, such as asthma, rhinitis, and dermatitis [2]. IL-4 promotes the immunoglobulin class switch from IgM to IgE [3]. IL-5 induces eosinophilia activity and infiltration, which is the critical response in the allergic asthma [4]. IL-13 acts to induce airway hyperresponsiveness (AHR) that contributes to atopic disease [5]. The suppression of Th2 responses is a feasible attempt to attenuate the symptoms of allergic asthma. Therefore, biologic targeted therapies have been developed to target the specific molecular pathways to treat asthma, especially those targets at IL-4, IL-5, IL-13, and IgE [6]. However, due to complex clinical symptoms and multiple mechanisms involved, the outcome of these trials has not been satisfied. Epidemiological studies indicate that patients often turn to complementary and alternative therapies, including dietary supplements [7]. Studies also showed that dietary oil, adlay, and medicinal herbs, such as Ganoderma and Andrographis, decreased Th2 cytokines productions and thus alleviated allergic responses in a murine model of asthma [8–12], suggesting the potential application of traditional herbal medicine for immunomodulation.

*Perilla frutescens *(Pf) leaf is a kind of aromatic vegetables, which is also used as the traditional medicine in Asia. Perilla has been demonstrated to exert antiobesity, anti-dyslipidemia, antioxidant, and anti-infammation [13–19]. Recently, some researches focused on its antiallergic effects [20–23]. The studies suggested water extract of Pf inhibited histamine release from mast cells [24]. Subcutaneous injection with perilla seed water/ethanol extract, as a traditional oriental therapeutic herbal acupuncture, seemed to reduce IgE, IL-4, IL-5, and IL-13 in BALF in OVA-induced asthma in mice [18].

However, they are either intraperitoneal injection [24], type II allergy evaluated by ear-passive cutaneous anaphylaxis or edema [20, 21], or lack of the important outcome of treatment in asthma, airway hyperresponsiveness (AHR) result [22]. There are few studies investigating the effects of oral supplementation of perilla extracts on overall allergic responses of asthmatic murine model. In addition, the crude polysaccharide isolated from Pf by hot water extraction was shown to increase nitric oxide and TNF*α* and IL-6 production both* in vitro* and* in vivo* [25]. Therefore, whether water or ethanol extracts of* Perilla frutescens *exert antiasthma effects* via* oral supplementation was investigated in this study using OVA-sensitized and challenged BALB/c mice.

## 2. Materials and Methods

### 2.1. Preparation of Extracts of* Perilla frutescens*

The crude extracts of* Perilla frutescens *(L.) Britt leaves were extracted as follows. Dried* Perilla frutescens *leaves were extracted with 5-fold (w/v) hot water for 60 min. The water extracts (PfW) were filtered through filter paper and concentrated using a rotary evaporator and then freeze-dried. The yield of PfW was 21.70% (w/w, dry basis). The ethanol extracts of dried* Perilla frutescens *leaves (PfE) were extracted with 15-fold (w/v) 95% ethanol for 24 hr. The PfE were filtered by filter paper and then evaporated in a rotary evaporator to remove the solvent. The yield of PfE was 9.22% (w/w, dry basis).

### 2.2. Animal Study, Sensitization, and Airway Challenge

Female BALB/c mice were purchased from the Animal Center at the National Taiwan University College of Medicine, Taipei, Taiwan, and maintained at the Department of Biochemical Science and Technology at the National Taiwan University. Animal care and handling conformed to accepted guideline [26].

**Figure 1:**
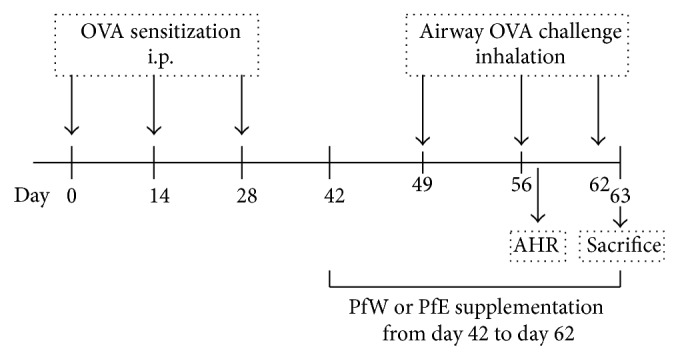
Schematic diagram of the experimental protocol. Eight-week-old female BALB/c mice were sensitized intraperitoneally (i.p.) by OVA for three times (10, 30, and 30 *μ*g/mouse) on days 0, 14, and 28. OVA-sensitized mice were challenged with 5% aerosolized OVA three times on days 49, 56, and 62. After 3 weeks of supplementation, mice were sacrificed and the BALF, serum, and splenocytes of mice were collected for further analysis.

The allergic asthma model, as well as the diet, was manipulated as described [11]. As shown in Figure 1, 8-week-old female BALB/c mice were sensitized intraperitoneally (i.p.) with 10 *μ*g ovalbumin (OVA; Sigma, St. Louis, Mo) in alum adjuvant (Imject Alum; Pierce, Rockford, IL). After three times OVA-sensitization, sensitized mice were grouped randomly into six groups for tube-feeding with 0 *μ*g (Control), 80 *μ*g (PfWL), or 320 *μ*g (PfWH) water extract or 80 *μ*g (PfEL) or 320 *μ*g (PfEH) ethanol extract of perilla leaves daily for 3 weeks. During these 3 weeks, the OVA-sensitized mice were challenged twice with 5% aerosolized OVA in PBS buffer by inhalation. In addition, mice without Pf supplement, that received alum without OVA, and that inhaled aerosolized PBS are used as the negative control group (PBS). After 3 weeks of Pf supplement, mice that received a third challenge were euthanized by CO_2_ inhalation and the BALF, serum, and splenocytes of mice were collected for further analysis.

### 2.3. Determination of Airway Hyperresponsiveness (AHR)

Airway function was measured by the whole-body plethysmography as described previously [11]. Twenty-four hours after the secondary challenge, AHR was measured when mice were stimulated with methacholine (Sigma) using whole-body plethysmography (Buxco, Wilmington, NC). Mice were placed in the main chamber of a whole-body plethysmography and challenged with aerosolized methacholine, increasing concentration from 12.5 to 50 mg/mL in PBS buffer. The AHR is expressed as the enhanced pause (Penh) values measured during each 3 min period.

### 2.4. Determination of Anti-OVA Antibodies

Serum anti-OVA IgE antibody titers were measured by ELISA method as previously described [27]. Briefly, 96-well plates were coated with 10 *μ*g OVA/mL NaHCO_3_ buffer. After overnight incubation at 4°C and being blocked with 1% bovine serum albumin (BSA)/PBS buffer for 2 hours at room temperature, the serum samples and positive control sera were appropriately diluted with blocking buffer and added to the 96-well plate. After 2 hours, the biotin-conjugated anti-mouse IgE (PharMingen, San Diego, CA) was added for 2 hours of incubation. Then, streptavidin-conjugated peroxidases and the enzyme substrate, 2,2′-azino-bis-3-ethyl-benzthiazoline-6-sulfonic acid (ABTS), were added and incubated for 20 min at room temperature. The antibody levels of the samples were compared with the positive control sera. The positive control was a pool of serum collected from OVA-sensitized mice with strong response (optical density > 1). The results of the antibody titer were expressed in ELISA units (EU), EU = (*A*_sample_ − *A*_blank_)/(*A*_positive_ − *A*_blank_).

### 2.5. Collection of BALF and Splenocytes

The BALF was collected with 5 instillations of 0.5 mL saline. Approximately 2.5 mL of fluid was recovered with each sample and the volume did not differ significantly among groups. The fluid was collected and kept at −80°C for eotaxin and histamine analysis. The cell pellet was resuspended in 250 *μ*L saline containing 10% BSA. The total cells were counted with a hemocytometer using the trypan blue dye exclusion method. BALF cells with a concentration of 2 × 10^5^ were cytocentrifuged and then stained with Liu's stain for eosinophil counts.

The splenocytes were prepared by aseptically removing spleens from sensitized and challenged BALB/c mice [27]. Splenocytes were counted with a hemocytometer using the trypan blue dye exclusion method. A concentration of 5 × 10^6^ cells/mL was cultured in 48-well plates in RPMI-1640 medium supplemented with TCM medium in the absence or presence of antigen, 50 and 100 *μ*g/mL OVA, for 48 hours. The supernatants in cell cultures were collected and stored at −80°C for cytokines analysis.

### 2.6. Cytokines Assay

The cytokine levels in splenocytes culture supernatants were measured by sandwich ELISA methods. Briefly, the anti-cytokine antibody was coated in the 96-well plates (Nunc, Roskilde, Denmark). After overnight incubation at 4°C and being blocked with 1% BSA/PBS buffer for 30 min, the samples and standards were added to the 96-well plates for 2 hours of incubation. The biotin-conjugated antibodies were added and incubated. After washing, the streptavidin-conjugated peroxidase was added for 1 hour. The substrate ABTS was added to each well for 20 min. The plates were read in a microplate autoreader (microplate autoreader; Bio-Tek Instrument, Inc. Winooski, VT) at 405 nm.

### 2.7. Eotaxin and Histamine Assay

The eotaxin concentration in BALF was determined by mouse eotaxin sandwich ELISA kit (R&D Systems, Minneapolis, MN). The eotaxin concentration was assayed according to the manufacturer's instructions. After the color reagent was added, the plate was incubated at room temperature for 20 min for color development. The absorbance was measured at 630 nm in the microplate autoreader. The eotaxin level in BALF was determined using the standard curve.

The histamine level was determined by Histamine-ELISA kit (IBL Hamburg, Germany). The manipulation was according to manufacturer's instructions for use. In brief, the culture supernatants and plasma standards were acylated with acylation reagent first. Then, aliquots of 50 *μ*L acylated sample and acylated standards were loaded into the 96-microplate wells, respectively. Aliquots of 50 *μ*L enzyme conjugate and 50 *μ*L antiserum were pipetted into each well to react for 3 hours. The plate was washed four times and 200 *μ*L tetramethylbenzidine (TMB) substrate solution was added for 30 min. Then the reaction was stopped by adding 100 *μ*L of stop solution into each well. The optical density was detected by the microplate autoreader at 450 nm. The histamine concentration is determined using the standard curve.

### 2.8. Statistical Analysis

Data were expressed as mean ± SD. The significance of difference between the Pf and the control groups was analyzed statistically by Student's *t*-test of the SAS program system (Strategic Application Software; SAS windows version 8.2, SAS Institute Inc., Cary, NC) throughout the study.

## 3. Results

### 3.1. Ethanol Extracts of* Perilla frutescens* Suppressed Th2 Responses of OVA-Sensitized Mice

**Figure 2:**
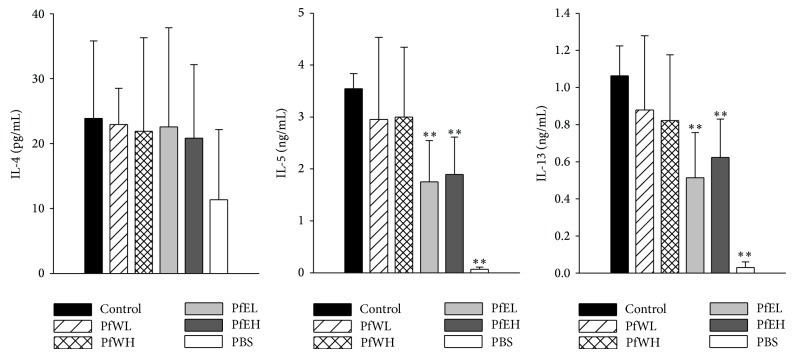
The Th2 cytokines produced by OVA-stimulated splenocytes from OVA-sensitized/challenged BALB/c mice supplemented with different extracts of* Perilla frutescens.* OVA-sensitized mice were fed with water (PfWL or PfWH) or ethanol (PfEL or PfEH) extracts of* Perilla frutescens* for 3 weeks, respectively. Splenocytes were isolated from OVA-sensitized/challenged mice and stimulated with OVA (100 *μ*g/mL for IL-4, 50 *μ*g/mL for IL-5 and IL-13) for 48 hours. The productions of Th2 cytokines in supernatant were determined by ELISA. Values represent mean ± SD, *n* = 7~8 for each OVA-sensitized group, and *n* = 6 for the PBS group as negative control. Statistical analysis was performed with Student's *t*-test, ^**∗**^ *P* < 0.05, ^**∗∗**^ *P* < 0.01 compared with the control group.

After feeding of two doses of water extract of Pf (PfWL and PfWH) or ethanol extract of Pf (PfEL and PfEH) for 3 weeks, splenocytes were isolated from OVA-sensitized mice and stimulated with OVA to examine the effects of PfW and PfE on allergen-specific Th2 responses. The Th1 cytokines IL-2 and IFN*γ* were not significantly affected (data not shown). However, Th2 cytokines IL-5 and IL-13 productions of OVA-stimulated splenocytes in the PfEL and the PfEH groups were significantly lower than those of the control group, as shown in Figure 2. The OVA-stimulated IL-4 productions were low and not affected by either water or ethanol extracts. In addition, PHA-stimulated IL-13 productions by splenocytes were also suppressed by PfE (data not shown). These data suggested that ethanol extracts of Pf could inhibit allergen-specific stimulated Th2 cells activity.

**Figure 3:**
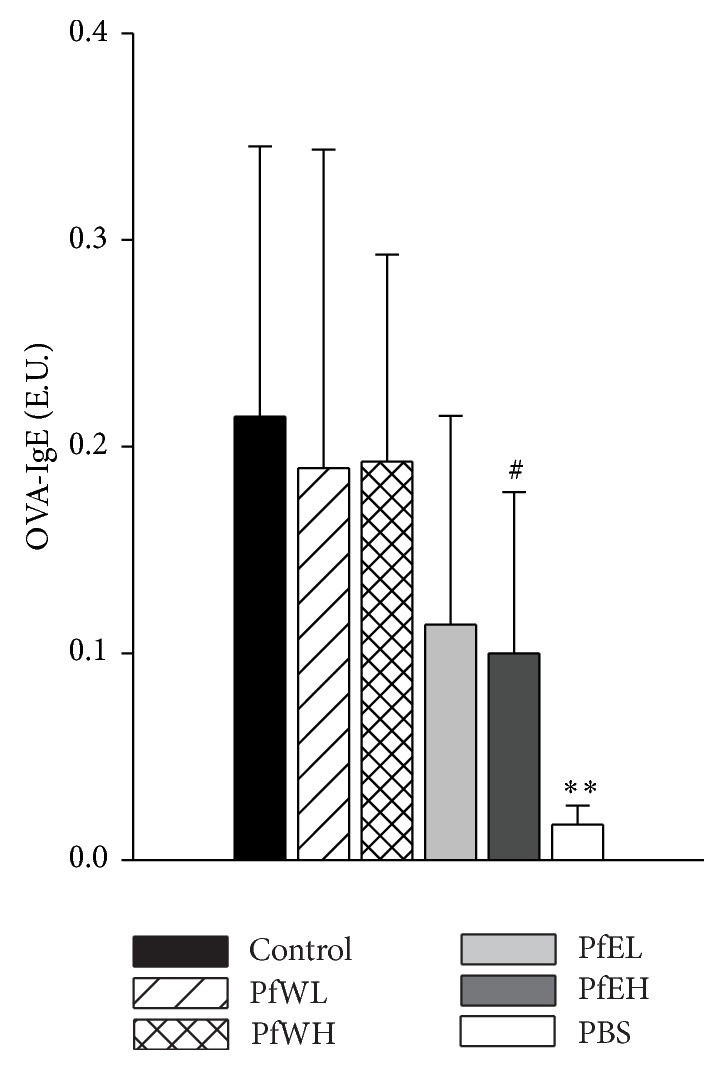
Serum levels of OVA-specific IgE in OVA-sensitized BALB/c mice supplemented with different extracts of* Perilla frutescens.* OVA-sensitized mice were fed with water or ethanol extracts of* Perilla frutescens* for 3 weeks, respectively. Sera of mice were collected and the OVA-IgE levels were determined by ELISA. Values represent mean ± SD, *n* = 7~8 for each OVA-sensitized group, and *n* = 5 for the PBS group as negative control. Statistical analysis was performed with Student's *t*-test, ^**∗∗**^ *P* < 0.01, ^#^0.05 < *P* < 0.1 compared with the control group.

The IgE antibody produced by Th2 cell-activated B cells tended to be lower in serum of OVA-sensitized mice supplemented with PfEH (Figure 3). The serum levels OVA-specific IgG_1_ and IgG_2a_ were not significantly affected (data not shown). This data suggested that ethanol extracts of* Perilla frutescens* might have potential to suppress serum IgE levels in OVA-sensitized mice.

### 3.2. Ethanol Extracts of* Perilla frutescens* Decrease Cells Infiltration in Airway Allergic Inflammation of OVA-Sensitized Mice

**Figure 4:**
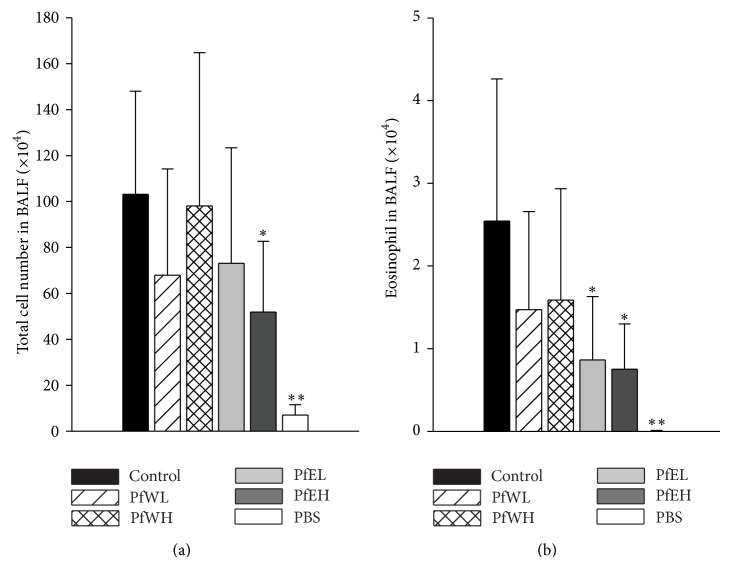
Total cells and eosinophils number in BALF of OVA-sensitized/challenged BALB/c mice supplemented with different extracts of* Perilla frutescens.* OVA-sensitized mice were fed with water or ethanol extracts of* Perilla frutescens* for 3 weeks, respectively, and BALF was collected after aerosolized OVA (50 mg/mL) inhalation challenge. Total cells (a) and eosinophils number (b) were determined. Values represent mean ± SD, *n* = 7~8 for each OVA-sensitized group, and *n* = 5 for the PBS group as negative control. Statistical analysis was performed with Student's *t*-test, ^**∗**^ *P* < 0.05, ^**∗∗**^ *P* < 0.01 compared with the control group.

The BALF was collected after OVA-inhalation challenge prior to sacrifice and the cell number in BALF was determined and shown in Figure 4. Few total cells and eosinophils were in the PBS negative control group. The infiltrated cells number increased after OVA-challenge as shown in the control group. The total cell number in BALF significantly decreased in asthmatic mice supplemented with PfEH (Figure 4(a)). Particularly, the number of eosinophils, the major cells contributing to airway inflammation of allergic asthma, was decreased by Pf supplement, significantly in both the PfEL and the PfEH groups (Figure 4(b)). These data suggested that* Perilla frutescens* inhibited the infiltration of inflammatory cells to BALF in asthmatic mice, especially the ethanol extracts.

**Figure 5:**
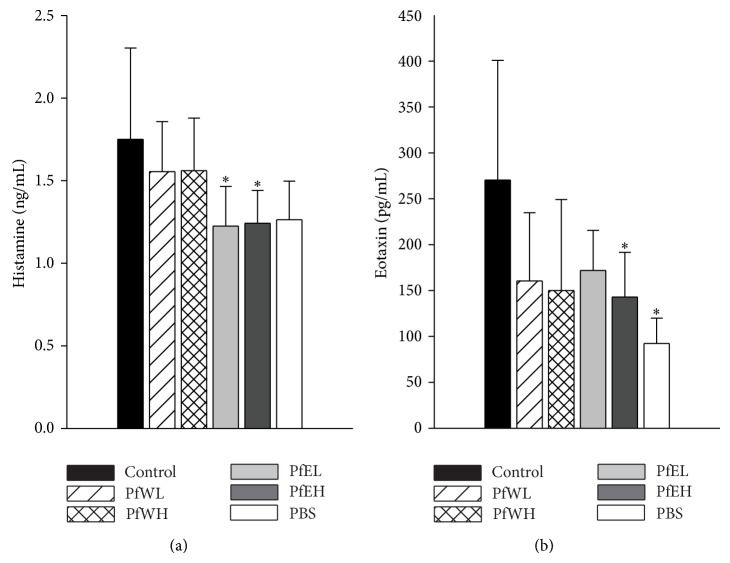
Histamine and eotaxin contents in BALF of OVA-sensitized/challenged BALB/c mice supplemented with the different extracts of* Perilla frutescens.* OVA-sensitized mice were fed with water or ethanol extracts of* Perilla frutescens* for 3 weeks, respectively, and BALF was collected after aerosolized OVA (50 mg/mL) inhalation challenge. The concentrations of histamine (a) and eotaxin (b) in BALF were determined by ELISA. Values represent mean ± SD, *n* = 7~8 for each OVA-sensitized group, and *n* = 5 for the PBS group as negative control. Statistical analysis was performed with Student's *t*-test, ^**∗**^ *P* < 0.05 compared with the control group.

The proinflammatory mediators such as histamine and eotaxin in BALF of OVA-sensitized mice were also suppressed by Pf, as shown in Figure 5. The histamine levels were significantly lower in both the PfEL and the PfEH groups. The eosinophil chemotactic protein eotaxin in BALF was also reduced in the PfEH group. Th2 cytokines in BALF of OVA-sensitized mice were also measured but were not found significantly lower by Pf in this experiment (data not shown). These results indicated that ethanol extracts of* Perilla frutescens* alleviated inflammatory cell infiltration and thus reduced the allergic inflammation in airway of OVA-sensitized and challenged mice.

### 3.3. Ethanol Extracts of* Perilla frutescens* Decrease Airway Hyperresponsiveness (AHR) of OVA-Sensitized Mice

**Figure 6:**
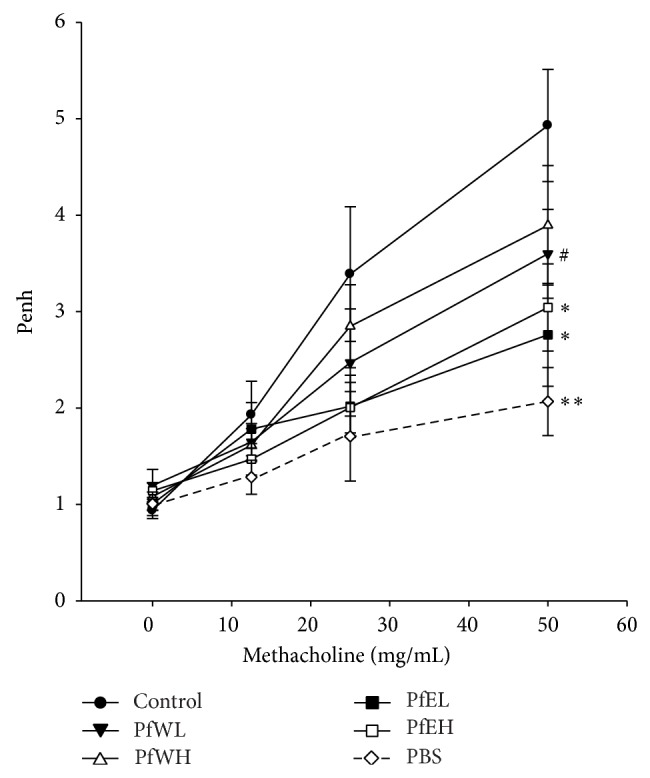
The AHR of OVA-sensitized/challenged BALB/c mice supplemented with different extracts of* Perilla frutescens.* OVA-sensitized mice were fed with water or ethanol extracts of* Perilla frutescens* for 3 weeks, respectively, and AHR was determined after aerosolized OVA (50 mg/mL) inhalation challenge. Values represent mean ± SEM, *n* = 7~8 for each OVA-sensitized group, and *n* = 5 for the PBS group as negative control. Statistical analysis was performed with Student's *t*-test, ^#^0.05 < *P* < 0.1, ^**∗**^ *P* < 0.05, ^**∗∗**^ *P* < 0.01 compared with the control group.

OVA-sensitized mice supplemented with two doses of water or ethanol extracts, respectively, were challenged with 50 mg/mL aerosolized OVA to further induce AHR. The results shown in Figure 6 demonstrated that the control group had significantly higher AHR, measured as Penh value, than the PBS negative control group after methacholine challenge. Ethanol extract of Pf significantly reduced AHR as significantly lower Penh values were detected in both the PfEL and the PfEH groups. Water extract of Pf tended to have low AHR (*P* < 0.1) at high dose (PfWH group) though the low dose PfWL group did not reach statistical significance. This data indicates that* Perilla frutescens* extracts possess inhibitory effects of airway hyperresponsiveness, possibly due to lower cell infiltration and inflammation in bronchiole and lung of allergic asthma.

## 4. Discussion

Allergic asthma is a chronic inflammatory disease in airway, which was induced by Th2-prone responses. The present allergic asthma murine model demonstrated that* Perilla frutescens* extracts, especially the ethanol extracts, decrease Th2 cytokines production, serum IgE level, cells infiltration, allergic mediator secretions, and AHR.

Early study examined the effects of different extracts of Pf on TNF*α* levels in inflammatory mice and found that water extracts had stronger inhibition than n-hexane or ethyl acetate extracts [28]. Water extract of Pf inhibited the histamine released from rat peritoneal mast cells* in vitro* [29]. Glycoprotein derived from the hot water extract of Pf demonstrated the effective component of* Perilla frutescens* Britton to inhibit mast cells degranulation [30]. These studies suggested the potential antiallergic effect of Pf. Further, our study showed that the ethanol extract of Pf had more effective inhibition than water extract on the histamine release in BALF of OVA-challenged mice. We suggested that this inhibition might be related to the suppression of Th2 activities.

It has been known that IL-4 plays a critical role in IgE production. IL-4 receptor was significantly associated with asthma risk [31, 32]. Allergen-stimulated IL-4 secretions by splenocytes from sensitized mice were low in this study and not suppressed by Pf, which might explain the reason why sera IgE levels were not significantly lowered by Pf. The tendency of lower serum IgE in the PfEH group might be partially due to lower Th2 activities such as allergen-stimulated IL-5 and IL-13 productions. IL-13 polymorphisms were consistently associated with asthma and serum IgE in asthma populations [33]. Our study showed that the ethanol extracts of Pf significantly decreased IL-5 and IL-13 productions from splenocytes of OVA-sensitized mice. Blocking the binding of IgE to its receptors is the most effective therapy strategy for allergic disease [34]. The association between IL-13 and IgE productions in asthma [35, 36] suggests that ethanol extracts of Pf suppressed IL-13 secretion and thus tended to reduce IgE production [37].

IL-13 induced the eotaxin release by airway epithelial cells* in vitro* [38]. Our data also indicated that ethanol extracts of Pf decreased eotaxin production in BALF, consistent with its lower allergen-induced IL-13 secretion. The inflammatory cells, such as eosinophils, recruited into airway are the major clinical manifestations in allergic asthma. IL-5 is best characterized for eosinophilia that dominates airway inflammation on allergic asthma [39]. When asthmatic patients were given anti-IL-5 (mepolizumab), bronchial mucosal eosinophils decreased [40]. Furthermore, eosinophils not only act as terminal effector cells but also act to actively amplify allergic responses by promoting Th2 cell immunity [41], indicating the close relationship between eosinophils and airway hyperresponsiveness [42]. Th2-cell-derived cytokine IL-5, together with eotaxin, plays the critical roles in the induction of airway hyperreactivity and the development of chronic airway wall remodeling [43]. Present report indicated that high dose of ethanol extracts of Pf inhibited eosinophils and eotaxin levels in BALF and also decreased AHR. These data suggested that ethanol extracts of Pf exert attenuate airway hyperresponsiveness and inflammation in allergic asthma through inhibition of eosinophils and proallergic mediators.

However, the percentage of eosinophil in total cell in BALF was low in this study. It may be due to the OVA-challenge protocol. Continuous daily inhalation or intranasally challenge with allergen may drive more eosinophils in BALF. Our previous studies showed ~10% eosinophils in BALF of mice when challenged with OVA inhalation every three days [11], but only ~3% eosinophils were counted with challenge every seven days. The majority of cell populations in this study were neutrophils/basophils, which were 54% in the control group and 35–45% in the Pf groups.

Study showed that water extracts of perilla leaves improve atopic dermatitis [20] and identified the active constituent to be luteolin [44]. Rosmarinic acid extracts in Pf strongly inhibited hexosaminidase release from RBL-2H3 mast cells [45] and were shown to decrease the neutrophils and eosinophils recruitment in nasal lavage fluid of seasonal allergic rhinoconjunctivitis patients [46]. Recent report revealed that 80% ethanol extracts of purple perilla leaves contains 8.47% (w/w) of rosmarinic acid which is the major phenolic acid in perilla and commonly found in aromatic plants [47]. Our data suggested that the water extracts of Pf attenuated AHR in OVA-sensitized mice, but they are less effective than ethanol extract. It may be due to the same doses of different extracts used in our study. Recent report indicated that ethanol extracts of Pf significantly decreased TNF*α* production in BALF from LPS-induced airway inflammation and suggested that phenylpropanoids may contribute to the inhibitory activity of ethanol extracts of Pf on the lung inflammatory response [48]. As* Perilla frutescent* leaves are aromatic vegetables and can be consumed raw, cooked, or pickled [49], their application is feasible and expectable. In our study, the most effective dose for decreasing Th2 responses and AHR is 80~320 mg PfE daily for mouse. According to the dose translation from animal to human [50], it corresponds to 1.5~6 g (~5~20 pieces) of fresh* Perilla frutescens* leaves daily for a 60 kg adult and 0.7~3 g (~2.5~10 pieces) for 20 kg child, respectively.

In conclusion, ethanol extracts of* Perilla frutescent* leaves could downregulate Th2 activities to secrete less IL-5 and IL-13 and thus lower serum IgE level when counting allergen challenge. The cell infiltration, particularly eosinophils, and proinflammatory mediators such as histamine and eotaxin in BALF were significantly suppressed. As a result, AHR is alleviated by the extracts of* Perilla frutescent* leaves, suggesting that* Perilla frutescent* leaves are a potential herbal medicine for immunomodulation.
